# Effects of Wood-Derived Biochar on Germination, Physiology, and Growth of European Beech (*Fagus sylvatica* L.) and Turkey Oak (*Quercus cerris* L.)

**DOI:** 10.3390/plants11233254

**Published:** 2022-11-26

**Authors:** Andrea Vannini, Michele Carbognani, Giorgio Chiari, T’ai G. W. Forte, Fabio Lumiero, Alessio Malcevschi, Margherita Rodolfi, Tommaso Ganino, Alessandro Petraglia

**Affiliations:** 1 Department of Chemistry, Life Sciences and Environmental Sustainability, University of Parma, Parco Area delle Scienze 11/A, 43124 Parma, Italy; 2 Department of Food and Drug, University of Parma, Parco Area delle Scienze 11/A, 43124 Parma, Italy; 3 National Research Council, Institute of BioEconomy (IBE-CNR), Via Madonna del Piano 10, 50019 Sesto Fiorentino, Italy

**Keywords:** broad-leaved forests, carbon sequestration strategies, carbon stock, climate change mitigation, forest trees, seeds germination, photosynthesis, plant growth

## Abstract

Biochar (BC) soil amendments could partially counteract soil carbon (C) stock decrease in broad-leaved forests in Italy; however, its effects on the growth of representative tree species—*Fagus sylvatica* L. and *Quercus cerris* L.—has not yet been addressed. We examine whether seed germination and growth of these species are affected by addition of BC obtained from deciduous broadleaf trees. Seeds were left to germinate in greenhouse conditions under three different BC amendments: 0% (control), 10% and 20% (*v*/*v*). Seedlings were then subjected to controlled conditions under the same BC percentage. Biochar effects on seed germination were assessed measuring germination time and percentage, while effects on photosynthesis were assessed using leaf chlorophyll content (mg/m^2^) and photosynthetic efficiency (F_V_/F_M_). Plant growth was estimated by recording leaf number, longest leaf length and plant height. Biochar treatments had no negative effects on germination and early growth stage of the two species. Positive effects were found on the chlorophyll content of both species (ca. +8%) regardless of the treatment and on the leaf number (+30%), leaf length (+14%) and plant height (+48%) of *Q. cerris* (only with 10% BC). Biochar applications seem, therefore, a suitable method for increasing broad-leaved forest C stock in Italy.

## 1. Introduction

Forest ecosystems play a fundamental role in the global carbon (C) cycle as well as in mitigating global atmospheric CO_2_ concentrations [1]. However, their stability is seriously threatened by changes in both land use and climate, producing overall negative effects on their C storage ability. In particular, land use changes such as deforestation can severely reduce soil C percentages, especially in surface layers [2] which are already threatened by intensive upstream wood removal [3]. Climate change, on the other hand, may affect forest C stock through the promotion of both biotic and abiotic disturbing agents [4]: pathogens and bark beetles in the case of the former; drought, wind, soil erosion, and fire in the case of the latter, whose synergetic interaction is predicted to result in gross C losses of ca. 503 Tg C in the current decade (from 2021 to 2030) [5]. Moreover, soil C content is strongly correlated with soil respiration (i.e., CO_2_ release caused by the action of soil microorganisms) which, in turn, is directly influenced by soil temperature [6]. There is, therefore, growing concern about the potential effect of rising temperatures on future forest soil C stock, especially in temperate deciduous forests [7] which are providers of numerous ecosystem services [8].

To overcome current and future C losses in forests, specific management strategies need to be worked out and applied [9], among the most important of which are the prevention of deforestation together with the promotion of afforestation and reforestation. Forest C stock can also be increased by improving soil C sequestration [10], a viable carbon dioxide removal (CDR) strategy [11] that can also be facilitated by spreading pyrolysis waste materials such as biochar (hereafter BC) on the forest floor [12]. BC represents the solid residue of anoxic thermal biomass degradation carried out for energy purposes [13]. During this process, biomass (e.g., wood) is converted into a carbon-rich product (>65%) characterized by high environmental stability [13,14,15] which, once spread onto forest soils, can be used to promote their C stock [16,17]. This is a strategy of particular interest when applied as part of a circular economy [18].

Forest soil BC addition, however, not only increases the C stock, but can also pro-mote tree growth [19], a key process in the enhancement of atmospheric CO_2_ sequestration and, in turn, of overall future forest C stock. Plant growth stimulation following BC application is the result of the ability of BC to improve soil chemical properties [20], including porosity, water holding capacity, aggregation, pH, and cation exchange capacity—all highly important properties, especially in the early stages of seedling development [21,22]. In a meta-analysis, Thomas and Gale [19] estimated that BC additions had the potential to increase tree biomass by ca. 41%, but differences were found across studies which depended on BC characteristics, tree species, application rate, climatic conditions, and study method (pot or field). In fact, plant responses may in some cases be negligible, even negative, following BC applications. For example, when high BC percentages were evaluated (% *v/v*), black locust plants (*Robinia pseudoacacia* L.) showed an increase in height and dry weight of both shoots and roots when exposed to a 1–2% BC application, while with a 5% BC amendment almost no growth-enhancing effects were found, though reductions in root diameter were observed [23]. In another study, Douglas fir plants (*Pseudotsuga menziesii* Mirb) showed reductions in both height and stem diameter following a 25–50% BC amendment with their photosynthetic rate being also reduced, but only up to their active growth period [17]. Changes in the amount of nutrients in soils amended with BC were also observed, mainly for P and K, while N showed clear but non-conforming trends in both studies. It should be noted that, although biochar derived from plant material is reported to contain lower amounts of heavy metals than other types such as those derived from poultry litter [24], both aforementioned studies agree that the lack of positive effects may be the result of the presence of potentially toxic elements or compounds (PTEs),such as polycyclic aromatic hydrocarbons (PAHs) within the pyrolysis products tested; the onset of adverse effects on Douglas fir, in particular, was also associated with excess water retention due to BC addition, together with an abrupt rise in soil pH.

Seed germination—the first step in the life cycle of a plant, starting with the imbibition of the seeds and ending with the radicle emerging from their coat—could also be affected by BC applications. Concerning tree species, only a few studies, however, have been carried out in this area. Of these, the majority indicated no negative effects, at least for a <5% BC application rate where both coniferous and deciduous trees were concerned [25,26], with the exception of the black locust where positive to null effects were found on seed germination [23].

Considering the above results, the effects of BC amendments on both tree growth and seed germination would seem to be both very difficult to predict and to generalize. Therefore, before planning BC applications, a case-by-case evaluation is necessary, especially in poorly studied forest ecosystems. This study focuses on two tree species, European beech (*Fagus sylvatica* L.) and Turkey oak (*Quercus cerris* L.), representing the most common species to be found in Italian broad-leaved forests where a future decrease in soil C stock is predicted [27]. While BC application could partially counteract this process, the study of the effect of this CDR strategy on seed germination and seedling establishment in relation to these two tree species has so far not been addressed. The only findings available regard responses to charcoal hearts [28], an aged BC that roughly corresponds to an amendment with 342 t/ha BC [29], i.e., slightly less than 30% BC (*v*/*v*). Only a marginally significant negative effect of charcoal hearts on seed germination for *F. sylvatica* and a positive effect on plant growth for *Q. cerris* was reported by Carrari et al. [28], thus suggesting that an amount of BC lower than 20% may be either irrelevant or even positive for germination and growth. Given the lack of data on these tree species, selecting the right application rate is therefore of considerable importance, since a wrong BC application rate may adversely affect their growth and, in turn, the future CO_2_ sequestration of these forests. In fact, in the case of Eucalyptus (*Eucalyptus urophylla* L.), application rates higher than their respective level of tolerance found to be equal to 7.5% BC generated reductions in some growth parameters, including leaf, root, and stem dry matter, as well as total dry matter and stem diameter [30].

For this purpose, the present study aims to experimentally examine whether seed germination and seedling growth of *F. sylvatica* and *Q. cerris* are affected by amendments with 10% and 20% BC obtained from the pyrolysis of wood from a deciduous broadleaf forest. The current study represents the first attempt at evaluating the potential use of BC to increase the C stock of Italian deciduous broadleaf forests without altering the development or productivity of the most frequently grown tree species.

## 2. Results

BC amendments did not affect either germination time or germination percentage for both investigated plant species (*p* > 0.05; Table 1). Plants of both species grown with BC showed a higher chlorophyll content when compared to controls (*p* < 0.05), with no differences being detected between the two amendment treatments (*p* > 0.05; Table 2). In contrast, no significant influence of BC was recorded for the functionality of photosystem II (F_V_/F_M_; *p* > 0.05; Table 2).

**Figure 1:**
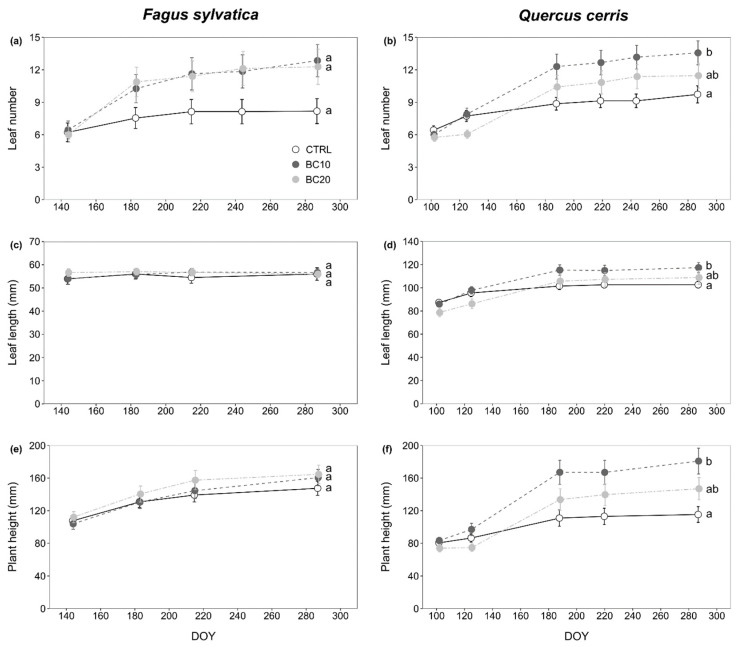
Morphometric parameters (mean ± standard error) over time (day of year, DOY) for *Fagus sylvatica* (**a**,**c**,**e**) and *Quercus cerris* (**b**,**d**,**f**) grown in soils amended with 0% (CTRL), 10% (BC10), and 20% (BC20); different letters indicate statistically significant differences between treatments (*p* < 0.05).

With regard to morphological responses, BC application enhanced the number of leaves, leaf length, and plant height only for *Q. cerris* and only as a consequence of the amendment with 10% BC (*p* < 0.05; Figure 1b,d,f); no effects were instead observed following the treatment with 20% BC when compared with the other two treatments, i.e., CTRL and BC10. Similarly, both the BC amendments did not change leaf number, leaf length, or height of *F. sylvatica* seedlings when compared to control samples (*p* > 0.05; Figure 1a,c,e).

## 3. Discussion

Results from this study highlight that relatively high BC application percentages can be considered as a safe strategy to increase C stock in Italian forest ecosystems dominated by deciduous broadleaf tree species, specifically European beech and Turkey oak. In fact, if BC is added to soil in percentages less than 20% (i.e., <260 t/ha or ca. 26 kg/m^2^), there are no observed negative effects on either early growth or development of *F. sylvatica* and *Q. cerris*. In particular, germination of both tree species was not compromised, nor were their physiological and growth/morphological responses altered. Interestingly, both tree species experienced an increase in their leaf chlorophyll content, independent of the BC percentage of application; in the case of *Q. cerris*, seedling growth/development was also enhanced, but only under 10% BC amendment.

Seed germination is the main physiological process involved in the maintenance and development of all existing plant species. Its preservation, therefore, is of paramount importance for maintaining the stability of any forest ecosystem. Deviations from normal soil characteristics, such as the presence of heavy metals (HMs) [31], can negatively affect germination, which is either slowed down or completely blocked. Due to its highly concentrated C structure and the high temperatures at which it is generated, BC can naturally contain both PTEs and compounds, such as HMs and polycyclic aromatic hydrocarbons (PAHs), already found to be the cause of reductions in germination in different plant species as reported in several studies [32,33,34]. Despite this, no factors inhibiting germination were observed for the two target tree species after their seeds were exposed to the tested BC, a result probably due to the very low concentration of such toxics within it. With regard to the concentration of toxic elements and compounds of human concern, the BC used in this study meets the most conservative European threshold certification limits for use in organic farming, i.e., 0.7 g/t dw for cadmium (Cd), 200 g/t dw for zinc (Zn), 70 g/t dw for copper (Cu), 25 g/t dw for nickel (Ni), and 4 g/t dw for the 16 Environmental Protection Agency PAHs [35]. The addition of biochar also generates increases in soil electrolytic conductivity (EC) [36]—a parameter known to influence plant development, especially those of agronomic interest [37]. Some types of biochar may be characterized by very high EC values, i.e., 1000 mS/m [38,39], a feature which could produce possible negative effects by reducing the germination of certain plant species [39]. Even though the biochar tested in this study shows an EC approximately 10 times higher than that of the soil used (ca. 20 mS/m, i.e., ca. 2 dS/m), this value would still appear to be within the range of limited effect on plant development [40]. The observed EC increases following BC addition (+90%; see Table S1) can, therefore, be considered almost entirely negligible or, in any case, ineffective in generating a potentially negative effect on seed germination of the two tree species tested.

As for photosynthesis—the main process for the development of any plant species—reductions in its functionality, and thus in the plant’s ability to produce both carbohydrates and energy, can limit plant development and growth, hindering a plant’s coping mechanisms in the face of natural and environmental stresses. Even though BC addition to soil, whether forest or agricultural, is undoubtedly an anthropogenic event, i.e., one that would not naturally occur in a forest, the negative effects of such applications on plant photosynthesis are extremely limited [36] and, for the most part, result in either positive or negligible effects. In detail, positive effects of BC amendments on plant photosynthesis were observed for spinach (*Spinacia oleracea* L.), velvetleaf (*Abutilon theophrasti* MediK.), purple willow (*Salix purpurea* L.), and maize (*Zea mays* var. ‘Amadeo’ DKC-3399) [41,42], whereas negligible effects were found for wheat (*Triticum aestivum* L. cv. Amaretto) and apple tree plants (not defined species and var.) [43,44,45]. In this study, BC amendments significantly enhanced leaf chlorophyll content for both species, irrespective of the application percentage, with an overall increase of ca. 9% for *F. sylvatica* and ca. 8% for *Q. cerris*. Such an increase following BC amendments could be related to increases in availability of nutrients such as nitrogen (N), phosphorous (P), potassium (K), calcium (Ca), and magnesium (Mg) [46,47,48] resulting from the fertilizing action of BC. In fact, as reported in Table S1, BC amendments contributed to increases in the content of available soil nutrients in proportion to the percentage of application, with a mean increase of 58% for N, 177% for P, 392% for K, 156% for Ca, and 52% for Mg when compared to control soil (BC0 samples). This increased nutrient availability not surprisingly results in an increased accumulation by the root system which, in this study, is highlighted by the reduced content of the aforementioned nutrients in the amended ‘growth soil’ (i.e., soil measured three months after planting) when compared to ‘control soil’ (freshly BC amended soil) (Table S1), with a mean of 189% for N, 34% for P, 63% for K, 42% for Ca and 28% for Mg. On a global scale, the amount of chlorophyll in forest plant leaves mainly depends on the characteristics of both soil and climate [49]. In a controlled environment, or in experimental sites, however, both these parameters are assumed to be stable, thus making a correlation between the chlorophyll content and the amount of N inside leaves easier, an amount which is dependent on N abundance in the growing media [50]. Although data relating to the amount of N inside the leaves is not available for this study, the percentage of N removed from the amended soils by the plants (189%) can be taken into account in order to hypothesize that the observed increase in the chlorophyll content in both tree species was caused by the increased availability of N in the growing soil following BC amendment—the effectiveness of BC in increasing both the content of N in the leaves and the amount of chlorophyll in plant species is, in fact, well-known [51,52]. The positive effect of biochar on increasing the availability of N for plants, thus also increasing the concentration of chlorophylls in their leaves has, in fact, been observed [53,54]. Furthermore, BC can promote the retention of N in the soil by reducing its volatilization as NH_4_^+^ and N_2_O, although its effectiveness seems to depend on the type of associated treatment [55,56].

Additionally, BC can promote nutrient assimilation by plants on account of its ability to change some soil chemical characteristics [57] such as cation exchange capacity (CEC) and pH. In detail, CEC soil enhancements improve the soil’s ability to retain positive ions (such as the aforementioned nutrients), which, in turn, become less prone to percolation loss and, therefore, more available for plant uptake [58]. On the other hand, increases in pH values due to the alkaline nature of BC [59] can lead to enhancements of N mineralization with positive effects on its availability to plants [60,61]. In line with this assumption, readily amended soils in the present study showed increases in both CEC and pH of 32% and 12%, respectively (Table S1). Moreover, the shift in soil pH from weakly acidic to basic may also have limited the mobility and thus the availability of toxic elements for plants [62,63].

Regarding photosynthetic efficiency (F_V_/F_M_), no changes were observed, suggesting no damage (or stimulation) to photosystem II functionality occurred for the investigated tree species. To the best of our knowledge, information on the effect of BC applications on the photosynthetic functionality of both European beech and Turkey oak is minimal, since the only available results originate from a study which used relic charcoal hearts [28]. The results of this study showed a negative (although negligible) effect on the photosynthetic performance of both tree species, which was explained by the low amount of P within the soil. Increases in chlorophyll content with no changes in the expression of photosynthetic efficiency, however, were observed for balsam fir leaves (*Abies balsamea* L.) exposed to an amendment with 75 t/ha of yellow pine BC [64] as well as for wheat plants (*Triticum aestivum* L. cv. Amaretto) grown in pots amended with 5% BC [44].

When turning to plant growth, BC amendments stimulated leaf number, leaf length, and plant height only in the case of *Q. cerris* and only under the lowest amendment percentage (10%), thus suggesting the presence of an optimum BC amount for the growth of this tree species under the investigated experimental conditions. In fact, the 20% BC amendment did not stimulate any of the abovementioned parameters when compared to control seedlings. Although information on the effect of fertilizer addition to the soil on *Q. cerris* growth is scarce, we can hypothesize that increases in plant biomass are due to the nutrient action generated by BC which, in our opinion, increased leaf length while simultaneously boosting the number of leaves and plant height—the correlation between leaf area and plant growth is in fact well established [65]. In line with our observations, growth of both pitch-pine (*Pinus rigida* Miller) [66] and Gmelin larch (*Larix gmelinii* (Rupr.) Kuzen [67]—an experiment conducted to evaluate the effect of the presence of ectomycorrhizae in BC soil amendments with regard to tree growth) was enhanced following soil amendments with 11% and 10% BC, respectively, while seedlings of hybrid poplar (*Populus nigra* L. × *Populus suaveolens* Fischer) exhibited negligible changes in their biomass following 25% BC amendments [68].

On the other hand, no positive effects on plant growth for European beech were detected, which could suggest either that an effective growth stimulation threshold for this plant had already been exceeded, or such a threshold had not yet been reached. Further studies on the evaluation of the effectiveness of application percentages from 1% to 70% (for example) may provide additional information on the behavior of this tree species, since other tree species showed negligible effects on their biomass stimulation between the range suggested above. In detail, negligible effects on growth were found for European spruce (*Picea abies* L.) from 15 to 60% BC [69] while Gmelin larch (*L. gmelinii*; a study conducted to assess the effect of pyrolysis temperature on BC characteristics and on plant growth) showed similar responses following exposure with BC percentages of 5% and 20% [70].

Overall, results from the current study showed no evidence of negative effects of BC amendments on either germination or growth of the two investigated tree species when applied in percentages less than 20%. However, mixing BC with the soil is a difficult task, given the practical and economic limitations of forest environments (plowing this soil is impossible). For this reason, the direct release of BC onto the forest surface would seem to be the most economically convenient and feasible method to increase forest floor C stock. When compared to soil mixing, this has actually been found to be the most efficient method for promoting plant growth [67]. Increasing plant growth determines an increase in forest biomass in both above—and belowground vegetative plant organs and this, together with the ability of forests to sequester more CO_2_ from the atmosphere, will ensure greater efficiency in the mitigation of climate change.

## 4. Materials and Methods

### 4.1. Description of Tree Species

The European beech (*F. sylvatica*) is the most widespread deciduous tree in Europe [71] with a native range covering woodlands from southern Scandinavia to southern Italy, as well as from western Turkey to northern Spain. In central Europe, this tree species is typical of lowland forests, whereas in Italy and Spain (the southern limits of its range) it represents a typical mountain species found exclusively above 1000 m a.s.l. [72]. In Italy, European beech occupies ca. 12% of the national forest cover, an area extending over 8,759,200 ha [73].

The Turkey oak (*Q. cerris*) is a deciduous tree native to southern Europe and Asia Minor, representing the dominant species in the mixed forests of the Mediterranean basin [74]. In Italy, this tree species is widespread, ranging from the plains to an elevation of approx. 1000 m a.s.l., occupying 10% of the abovementioned national forest area [73].

### 4.2. Seed and Soil Collection

In October 2020, seeds of *F. sylvatica* and *Q. cerris* (ca. 500 for each species) were collected from a forest area in the Tuscan-Emilian Apennines, located between 800 and 1000 m a.s.l. (44°23′59.7″ N, 10°02′08.5″ E). Seeds of *F. sylvatica* were harvested directly from trees, while those of *Q. cerris* were collected from the ground. In both cases, seeds were collected from 20 different plants located at a minimum distance of 500 m from each other. In December 2020, and from the same site, soil samples (up to 30 cm depth, avoiding litter) were also collected.

### 4.3. Experiment Design

In the laboratory, the entire soil pool was divided into three homogeneous soil sets that were then separately amended with three different volumes of BC (*v/v*) in order to obtain the following three BC treatments: 0% (CTRL), 10% (BC10), and 20% (BC20). When the average density of forest soils in Emilia-Romagna (ca. 1.3 t/m^3^) [75] was taken into consideration, the selected BC percentages corresponded to soil amendments of 0 t/ha, 130 t/ha, and 260 t/ha, respectively. The amended soils, together with soil used as a control treatment, were then used to fill 813 pots of 0.64 L (8 cm × 8 cm × 10 cm), with a total of 271 pots prepared for each amendment. Biochar was produced from woody biomass derived from the management of deciduous broadleaf forest located in the Tuscan-Emilian Apennines, using a 125 kWe industrial gasifier (500–650 °C; Holz Energie); BC chemical characteristics are presented in Table 3.

The chemical and physical characteristics of soils without (i.e., soil only) and immediately after BC soil amendment are reported in Table S1 (see ‘Control soils’).

Before seeds were transferred to pots, beech seeds were subjected to 30 days of cold stratification (4 °C) while those of Turkey oak received none. In December 2020, one seed per pot of either *F. sylvatica* or *Q. cerris* was planted, with a total of 135 and 136 seeds for European beech and Turkey oak, respectively, for each treatment. All pots were then placed in an outdoor greenhouse under natural light until germination was completed, i.e., two weeks after no new germinated seeds were observed (April 2021). Pot position followed a block design consisting of groups (statistical replicates) made of four to 14 pots (pseudo-replicates). Seedlings characterized by similar height were then transplanted into new pots of 1.7 L (10 cm × 10 cm × 17 cm) containing the same soil composition as that reported in the first step. The specific number of seedlings selected for each treatment is provided in Table 4.

Transplanted plants were then transferred into a climatic chamber with a temperature of 22 °C, a relative air humidity of 73%, a photosynthetic photon flux density of 170 µmol/m^2^/s and a day/night cycle of 16/8 h for three months (i.e., April–October 2021). In order to reduce the influence of microclimatic conditions on plant growth, pot positions were changed weekly. All plants were watered weekly with 100 mL deionized water.

#### 4.3.1. Seed Germination

For each treatment, seed germination results were reported as germination time and germination percentage, calculated as follows:

Germination time (days) = mean germination time for each treatment

Germination % = (number of germinated seeds/number of total seeds) × 100

Seeds were considered as germinated only after cotyledon leaves were visible. Germination assessment was carried out every two–three days and the percentage of seeds germinated for each treatment was calculated at the end of the greenhouse experiment period. Nine replicates per treatment were used for European beech and seventeen for Turkey oak.

#### 4.3.2. Physiological Measurements

Plant physiological measurements were carried out by means of two photosynthetic indicators: chlorophyll content and chlorophyll *a* fluorescence, both widely used to assess the effects of biotic and abiotic constraints on plants [76,77] including heavy metal contamination [78]. Chlorophyll content, expressed as mg/m^2^, was measured using a chlorophyll meter (atLeaf+; Wilmington, DE, USA), with records subsequently converted into chlorophyll concentrations by applying a specific conversion factor officially provided by the manufacturer [79] to each value. At the end of the experiment (August 2021), two leaves were randomly selected for each plant and three measurements per leaf were taken (the average of the two means per leaf was then considered as a statistical replication). Regarding photosynthetic efficiency (F_V_/F_M_), which is a well-known indicator for assessing the PSII functionality [80], this was measured using a plant efficiency analyzer (Handy PEA; Hansatech Ltd., Norfolk, UK). Prior to each measurement, one randomly selected leaf per plant was dark-adapted for 40 min and then lit using an actinic light (3000 μmol photons m^−2^ s^−1^) for one second, after which F_V_/F_M_ was recorded. Physiological measurements were taken from 16 April to 14 October (considered as the end of the first year of growth), but only those recorded in August for the European beech and in July for the Turkey oak were reported. During these two selected periods, both plants show their peak photosynthetic system activity.

#### 4.3.3. Morphometric Measurements

Changes in morphometric parameters following the treatments were assessed by means of plant height (mm), leaf length (mm), and leaf number. Plant height was measured as the distance between the soil pot level and the topmost bud. Leaf length was measured only for the longest leaf of each seedling. The number of leaves only applied to unfolded leaves. Morphometric parameters were monitored over time as shown in Table 5.

### 4.4. Statistical Analysis

Statistically significant differences between treatments were evaluated by means of the pairwise permutation t-test using the Benjamini–Hochberg correction for multiple comparisons. All calculations were run using R software [81]. The presence of statistically significant differences in chlorophyll content between the three treatments was assessed using the original atLeaf data rather than derived concentrations, as values <16 cannot be converted to mg/m^2^. Concerning seeds germination and morphological parameters, statistical analysis was progressed only on the last day of measurement. Instead, concerning physiological parameters, only those recorded in August for the European beech and in July for the Turkey oak were statistically analyzed (for details, see Section 4.3.2. Physiological measurements).

## 5. Conclusions

The application of BC produced from deciduous broadleaf trees did not affect seed germination or plant growth of European beech (*F. sylvatica*) and Turkey oak (*Q. cerris*), two of the most common tree species in Italian broadleaf forests. BC amendments generated positive effects on the chlorophyll content of both species, irrespective of the treatment (ca. 8%), as well as on leaf number (30%), leaf length (14%), and plant height (48%) of *Q. cerris*, but only following the 10% amendment. No effect, either positive or negative, was observed for *F. sylvatica*, with further analyses of soil and plant nutrient components required to explain the absence of the effect of BC amendments on this species. In conclusion, until further studies on the impact of BC on other components of this ecosystem are carried out, BC amendment appears to be a viable solution for increasing C stock in broad-leaved forests in Italy.

## Figures and Tables

**Table 1:** Germination time and germination percentage (mean ± standard error) of seeds of *Fagus sylvatica* and *Quercus cerris* planted in soils amended with 0% (CTRL), 10% (BC10), and 20% (BC20) of BC; for each species, different letters indicate statistically significant differences between treatments (*p* < 0.05).

Parameter	Treatment	*Fagus sylvatica*	*Quercus cerris*
Germination time (days)	CTRL	59 ± 4 a	36 ± 1.7 a
	BC10	70 ± 3 a	38 ± 1.9 a
	BC20	61 ± 3 a	36 ± 1.5 a
Germination %	CTRL	21 ± 4 a	42 ± 5 a
	BC10	27 ± 3 a	49 ± 4 a
	BC20	27 ± 3 a	40 ± 5 a

**Table 2:** Expression of the physiological parameters (mean ± standard error) for plants of *Fagus sylvatica* and *Quercus cerris* grown in soils amended with 0% (CTRL), 10% (BC10), and 20% (BC20) of BC; for each species, different letters indicate statistically significant differences between treatments (*p* < 0.05).

Parameter	Treatment	*Fagus sylvatica*	*Quercus cerris*
Chlorophyll Index (mg/m^2^)	CTRL	41(251) ± 1.0 a	46(320) ± 0.5 a
	BC10	44(291) ± 0.8 b	49(360) ± 0.4 b
	BC20	45(301) ± 0.9 b	50(368) ± 0.5 b
F_V_/F_M_ ^1^	CTRL	0.73 ± 0.01 a	0.80 ± 0.01 a
	BC10	0.72 ± 0.02 a	0.81 ± 0.00 a
	BC20	0.72 ± 0.02 a	0.81 ± 0.00 a

^1^ F_V_/F_M_ = photosynthetic efficiency of the photosystem II.

**Table 3:** Mean values of chemical analysis of the BC produced from wood derived from deciduous trees. Abbreviations: P_2_O_5_: assimilable phosphorus; K_2_O: available potassium; EC: electrical conductivity; Part. size (%): particulate size %, i.e., percentage of biochar for the respective through-fraction (mm); LOD: limit of detection; Σ PAHs: polycyclic aromatic hydrocarbons content sum.

Parameter	Value
pH	8.5
Ca/Mg	4.7
Mg/K	0.2
P_2_O_5_ (ppm)	734.9
K_2_O (ppm)	11,764.1
Ca^2+^ (ppm)	4843.2
Mg^2+^ (ppm)	628.4
Na^+^ (ppm)	107
EC (mS/m)	236
Surface area (m^2^/g)	213.3
Part. size (%) < 5 mm	90
Part. size (%) < 2 mm	70
Part. size (%) < 0.5 mm	24
Metals (mg/kg)	
Cadmium	<LOD
Zinc	<LOD
Copper	0.007
Iron	0.005
Nickel	<LOD
PAHs (mg/kg)	
Σ PAHs	3.7

**Table 4:** Total seedling number (statistical replicates) for *Fagus sylvatica* and *Quercus cerris* for each treatment: CTRL (control), BC10 (10% BC), and BC20 (20% BC).

No. Samples	*Fagus sylvatica*	*Quercus cerris*
CTRL	22	20
BC10	27	24
BC20	25	30

**Table 5:** Days on which the respective morphological parameters of *Fagus sylvatica* and *Quercus cerris* were measured.

Parameter	*Fagus sylvatica*	*Quercus cerris*
Number of leaves	−	12 April
	24 May	5 May
	2 July	7 June
	3 August	8 July
	1 September	1 September
	14 October	14 October
Leaf length and plant height	−	12 April
	24 May	5 May
	2 July	7 June
	3 August	9 July
	14 October	14 October

## Data Availability

The data presented in this study are available on request from the corresponding author.

## Supplementary Materials

The following supporting information can be downloaded at: https://www.mdpi.com/article/10.3390/plants11233254/s1, Table S1: Chemical parameters and analysis of soils amended with 0% (CTRL), 10% (BC10), and 20% (BC20) (*v*/*v*) of biochar immediately after the amendment (Control soils) and three months after plant growth (Growth soils). Reference [82] are cited in Supplementary Materials File.
