# Supplementary material for: Effects of Wood-Derived Biochar on Germination, Physiology, and Growth of European Beech (Fagus sylvatica L.) and Turkey Oak (Quercus cerris L.)
Source: Plants (Basel). 2022 Nov 26;11(23):3254. doi: 10.3390/plants11233254 (PMC9741182; doi:10.3390/plants11233254)
Supplement: Supplementary file 1 [file plants-11-03254-s001.zip › plants-2012932-supplementary.pdf]

Supplementary Material

Effects of biochar amendments on germination, physiology, and growth of European beech (*Fagus sylvatica* L.) and Turkey oak (*Quercus cerris* L.)

Andrea Vannini [1], Michele Carbognani [1], Giorgio Chiari [1], T'ai Gladys Whittingham Forte [1,*], Fabio Lumiero [1], Alessio Malcevschi [1], Margherita Rodolfi [2], Tommaso Ganino [2,3,+], Alessandro Petraglia [1,+]

[1] Department of Chemistry, Life Sciences and Environmental Sustainability, University of Parma, Parco Area delle Scienze 11/A, 43124 Parma, Italy

[2] Department of Food and Drug, University of Parma, Parco Area delle Scienze 11/A, 43124 Parma, Italy

[3] National Research Council, Institute of BioEconomy (IBE-CNR), via Madonna del Piano, 10-50019 Sesto Fiorentino, Firenze, Italy

*Corresponding author: tai.forte@unipr.it

[+]These authors have contributed equally to this work

S1. Soil analyses

To obtain information on the effect of biochar amendments on the chemical-physical characteristics of amended soils and their behaviour following plant growth, soils were analysed for their chemical parameters and essential element content. Measurements were carried out immediately after the amendment and at the end of the entire plant growth cycle (Table S1). The following is a brief description of the methods used. **Soil pH:** Samples (fraction <2mm) were stirred for two hours in distilled water in the ratio 1:2.5; measurements were taken after samples were centrifuged and filtered at 0.45um. **Total limestone:** Gas-volumetric determination of CO_2 was recorded by treating a fine soil sample (fraction <2mm) with HCl (1:1 *v/v*). **Organic Carbon (Walkley-Black method):** Organic carbon was converted into CO_2 using a solution of potassium dichromate in the presence of sulphuric acid, and its subsequent volumetric titration with a Fe (II) sulphate heptahydrate ($FeSO_4$ - $7H_2O$) 0.5 M solution. **Total N (Kjeldahl Method):** Ammonia nitrogen was distilled in an alkaline medium and absorbed in a solution of sulphuric acid (0.01 M). The excess sulphuric acid was then titrated with a solution of sodium hydroxide (0.02 M); bromocresol green ($C_{21}H_{14}O_5Br_4S$) and methyl red ($C_{15}H_{15}N_3O_2$) were used as indicators. **Exchangeable Ca, Mg, and K:** The fraction of exchangeable Ca, Mg, and K was first removed from the exchange sites in the soil using a solution of $BaCl_2$ and triethanolamine (buffered at pH 8.2 with hydrochloric acid) and then analysed by flame atomic absorption spectrophotometry (FAAS). **Assimilable phosphorus (P_2O_5; Olsen method):** Phosphorus content was determined spectrophotometrically using the ascorbic acid method. The determination was performed colorimetrically by reading the sample at 882 nm. **Organic matter (%):** Organic matter content was estimated by multiplying the organic carbon content by 1.724. **Electrical conductivity**: Samples (fraction <2mm) were stirred for 2 hours in distilled water in the ratio 1:5; readings were taken after the sample was centrifuged and filtered at 0.45um. All methods are fully described in the Official Journal of the Italian Republic, General Series No. 248 of 21/10/1999 of the Ministry of Agriculture and Forestry [82].

Table S1. Chemical parameters and analysis of soils amended with 0% (CTRL), 10% (BC10), and 20% (BC20) (v/v) of biochar immediately after the amendment (Control soils) and three months after plant growth (Growth soils); growth soils correspond to those in which only *Q. cerris* was cultivated. Abbreviations: P_2O_5: assimilable phosphorus; K_2O: available potassium; CEC: cation exchange capacity, EC: Electrical conductivity; nda: no data available.

Parameter	Unit	Control soils			Growth soils		
		CTRL	BC10	BC20	CTRL	BC10	BC20
pH	-	4.8	6.4	7.1	5.6	7.3	7.5
Total limestone	%	3.1	nda	6.4	4.0	7.9	9.6
Organic C	%	5.4	8.3	7.4	2.8	2.5	3.1
C/N	-	11.6	12.5	11.0	8.8	10.7	16.0
Ca/Mg	-	7.4	11.4	13.6	6.7	10.7	12.0
Mg/K	-	4.6	2.8	1.0	23.6	4.3	1.3
N tot	g/kg	4.2	6.6	6.7	3.2	2.5	2.1
P_2O_5	ppm	21.3	42.9	75.3	9.0	33.9	54.5
K_2O	ppm	147.4	368.1	1019.5	39.3	195.1	701.4
Organic Matter	%	9.3	14.3	12.7	4.9	4.3	5.3
CEC	mwq/100g	28.4	39.2	35.3	19.8	21.1	23.2
Ca^{2+}	ppm	2150.8	4994.8	6012.4	2565.9	3768.7	3991.1
Mg^{2+}	ppm	175.3	265.4	269.1	233.3	215.8	201.4
Na^{+}	ppm	14.5	18.6	15.3	12.5	16.5	16.7
EC	mS/m	20.1	30.8	45.8	nda	nda	nda
